# Hydrophobia

**Published:** 1872-09

**Authors:** John C. Dalton

**Affiliations:** New York


*Hydrophobia.* A Lecture delivered at the Medical College of the Pacific. By Prof. JOHN C. DALTON, M.D., of New York. (Reported by Henry Gibbons, Jr., M.D.)

The study of hydrophobia is invested with unusual interest from several circumstances. First, from its communicability; second, from its extraordinary, probably universal, fatality, there being no authentic case of recovery; and lastly, from the terrible character of its symptoms.

It is an extremely rare disease. Many practitioners have never seen a case; and yet it does recur with tolerable regularity. During the six years from 1862 to 1867, inclusive, a hundred and seven cases occurred in France. Taking the average population of that country as thirty-six millions, this would give one case every two years for each million of inhabitants. But the disease prevails to a greater extent in cities, for obvious reasons. The denser population of the city brings the rabid animal in almost certain contact with many persons, while in the country he may die before meeting any one to bite. Again, the statistics of cities are more accurate. In the department of the Seine, including Paris, the population averaging one million, there was, for the twenty years from 1842 to 1862, an average of two and one-third cases in each year. In New York, for the six years from 1866 to 1871, there were twenty-two cases, an average of two and two-thirds each year. Taking all these statistics together, we judge that we have, generally speaking, three cases of hydrophobia a year to every million of people.

Another point of great interest is the frequency with which hydrophobia follows the bite of the animal. In the neighborhood of Paris is the Veterinary School of Alfort, where this subject has been fully investigated. It was found that of dogs bitten by rabid dogs, but one in three becomes rabid. In the human race the proportion is much less. John Hunter estimated it at only one in twenty-one. This has been considered far too low; but it has received singular confirmation in the investigations made at the veterinary school of Alfort. In the Department of the Seine, a hundred cases of hydrophobia in dogs occur annually. It was found that of twenty-five of these, ten only bit persons, and these ten bit fifteen persons. At this same rate the hundred dogs would have bitten sixty persons. But we have already seen that only between two and three cases of hydrophobia in man occur annually, hence we arrive at almost the same result as Hunter.

The less susceptibility of man to hydrophobia is owing to a variety of causes. Man is undoubtedly less susceptible to the poison. Besides, there are a great many physical causes operating. It is not the bite, but the saliva that poisons. If a man be bitten, it is usually through the clothing, or the boot, and the saliva is wiped off, or should the saliva touch the wound, it is often removed by the action of the person, by squeezing or washing or rubbing the wound, or if there be much bleeding, the blood may wash away the poison before it has had time to be absorbed. But if absorption have taken place, it is absolutely certain to produce death.

Now in regard to the signs which follow a bite. There are no immediate signs. The mad dog does not display any ferocity. It may be a chance bite, a snapping of the jaws without the intention of biting, that brings the poison in contact with the raw surface. Indeed, the dog may not bite at all, but the saliva touch a wound or abrasion or sore already existing. The bite is generally not large, and heals well. Then comes the period of incubation, such as occurs in small-pox, measles and scarlatina. The person is inoculated, but the poison is not yet diffused over the system. This period of incubation varies. It is rarely less than a month; it is often delayed to two or three, and may extend to the end of the sixth month; and there have been cases in which the disease has not exhibited itself until a year has elapsed. There are stories of longer periods, even to eight years; but these are incredible, particularly as during that time there are so many chances that another bite may have been received. There are then no symptoms for a month. Each succeeding week of exemption after the month has elapsed diminishes the liability. At three months the patient is probably safe; at six months almost certainly so; but what weeks of extreme anxiety must he experience before the danger is past!

If hydrophobia is about to occur, there is generally slight irritation about the wound; an itching, possibly swelling, with red lines extending therefrom; but this is not always the case. Next follows stiffness of the neck; then febrile action, with loss of appetite, succeeded by difficulty in moving the jaw and difficult deglutition; then occurs a most remarkable symptom—a vague and unaccountable anxiety, not of any special thing, and quite unexplainable. After from six to twelve hours, this amounts to delirium. Finally, hallucinations occur, and the friends are obliged to restrain the patient by force. This necessity for restraint has given rise to the popular idea that hydrophobia induces a tendency to attack persons. It is, however, entirely erroneous. Another symptom has given rise to the popular impression that there is a great dread of water; hence, indeed, the name of the disease. But this is equally erroneous. There is great difficulty in swallowing, whether water or other things. An attempt to do so brings on convulsive constriction of the glottis, accompanied by great pain and distress, which any one who has inhaled chlorine in the laboratory has experienced. In this respect hydrophobia is very similar to tetanus. In that disease, a breath of air, the moving of the bed clothing, or some such slight circumstance, will cause sudden reflex convulsions of opisthotonos. So with swallowing in hydrophobia. After repeated trials, so painful, he gives up in despair; he throws the water from him and will not again attempt to swallow. When this condition is reached, the patient has not long to live. He dies of extreme nervous depression and exhaustion, generally in forty-eight hours, though he may live three days.

What treatment can be instituted in these cases? Of course you will think of opium, of chloroform, and other narcotics. They are useless. There is only one thing to be done, and that at the very first: Cauterize. Some have recommended to cut out the wound first and then cauterize. I do not see the use of this. We cannot tell the depth of the wound. We cannot be sure of preventing the knife from cutting into the bitten portion, in which event every movement of it would carry the poison farther. Use the caustic freely. Lunar caustic is the best, being more readily obtainable than any other; and its stick form renders it easy of application. Apply it thoroughly until every portion of the wound has lost its sensibility. Generally speaking, if this is done early enough, it is efficacious.

But there is something of a great deal more importance than this. Barbarous as it may appear, it is not so important to save life as it is to prevent a dog with hydrophobia from being at large, with the probability of his biting.

Although the proportion of those who have hydrophobia to those bitten is so small, I do not know that the majority of those bitten would not prefer speedy death to the terrible anxiety of uncertainty. But how shall biting be prevented? Only know that the dog is mad. Fatal bites result because the disease is not recognized. To distinguish hydrophobia in the dog is vastly more important than to distinguish it in man, and there are signs by which it may be distinguished before the tendency to bite is exhibited.

Now of the signs of hydrophobia in the dog. I presume that ninety-nine of every hundred, perhaps nine hundred and ninety-nine of every thousand, think that dogs get mad only in hot weather—never in cold—and that they have a horror of water. This is not true. The disease is no more prevalent in hot than in cold weather. Dogs have no horror of water, but being feverish, drink frequently. The symptoms in the dog have been fully studied at Alfort. He first gives evidence of being sick, loses his appetite, refuses food, skulks out of sight, avoids his companions and playfellows, and hides in dark corners. Now, as hydrophobia is so dangerous a disease, a dog showing signs of sickness should be watched. Whatever the nature of the sickness may be, however, a dog will always act in this way. These symptoms are not pathognomonic. But there is another symptom which, if it occur in connection with these, is very suggestive. It is a peculiar agitation. The dog with hydrophobia does not lie in one corner ten minutes together. He is not satisfied to remain in one locality. Should his master call him he goes to him, but quickly returns to a corner. If he has a kennel he runs into it. He piles the straw up in the middle of the kennel and lies upon it. In a few moments more he jumps up and pushes all the straw out on the ground. These are extremely suspicious circumstances, and call for careful confinement of the dog.

Next, the dog has hallucinations. All at once in changing his position he stops, pricks up his ears, and seems to hear something. This is but momentary. Again, he appears to hear some other dog on the opposite side of a wall or a door, and scratches to get to him. He snaps at imaginary flies. Now is surely the time to place him in confinement. Up to this period he has shown no disposition to bite. It must be borne in mind that when sick, the natural disposition of the dog remains. If he be naturally excitable and aggressive, he will be more apt to bite. He will be more apt to bite a stranger or a beggar than one of the family, or his master. Indeed, he will not bite his master, unless driven too far. He will even come, though reluctantly, at his master's call, and will perhaps receive a whip or two before his unusual excitability overcomes his habitual control and induces him to bite. All this time the hydrophobiac dog will drink. He will take more water than usual; his apparent repugnance to it never being so strong as in the human subject.

The next symptom is a depraved and unnatural appetite, exhibited by biting furniture, knawing curtains or the carpet, swallowing bits of wood, coal, brick, even his own dung. This adds much to the belief that he has hydrophobia. All know that some pet dogs have a habit of knawing books, but hydrophobiac dogs, having no such habits, do the same, and, besides, swallow what they knaw off.

We have, then, agitation and extreme restlessness, hallucinations, and depraved appetite, as symptoms of the approach of the dangerous stage of hydrophobia. The following incident will illustrate the importance of distinguishing the disease at this early stage, and the value of these symptoms: Two ladies, with the little daughter of one of them, lived together. They had a little pet dog, which became ill, and hiring a carriage they drove out to Alfort to consult the veterinary surgeon. It was after visiting hours, and not being able to see him, they returned the next day. The surgeon was now able to decide at once, from their account, that the dog had hydrophobia. He learned that the dog had developed a disposition to tear the curtains, and had not slept during the whole night, but was heard pattering over the floor. Unfortunately, in the interval between the two visits, he had bitten the little girl.

When a member of a family is supposed to have been bitten by a rabid dog, it is the habit to kill the dog at once. This is all wrong. The only means of deciding whether the supposition is correct is thus destroyed. It is well known that the house-dog is prone to epilepsy. If in the paroxysm he may have accidentally bitten or appeared to snap at some one, let him be confined and watched for a few days.

The fourth symptom is a peculiar bark. I have never heard it. It does not always occur, but when it does exist, is considered a very valuable symptom. It is very difficult to describe, but is spoken of as a loud, quick bark, followed by a series of diminishing howls.

The fifth symptom is insalivation, in regard to which a misconception has arisen. There is no great abundance of saliva, but it is peculiar in quality. It is very viscid, and hangs down in strings from the mouth, giving to the human and the dog an appearance of peculiar and unaccountable distress. The dog appears to be annoyed beyond expression, and makes every effort to remove the viscid strings of saliva with his paws. These efforts have occasionally given rise to the idea that a bone or piece of wood is lodged in the teeth or throat, and in one instance a veterinary surgeon, in making an examination, was bitten.

Now the aggressive disposition begins to show itself. The insane desire to bite is first excited by animals of its own species. Next, the dog attacks man, but even now discriminates in favor of its master and friends. A stranger or some one coming to the door is attacked. Now that the disease is fully established, the dog disappears from home, probably with the idea of seeking a deserted place and withdrawing from observation. But new and greater sources of irritation meet him at every step. The noise of carts and horses and of people increases his excitability. If he should escape death and succeed in reaching the fields, he reels along from side to side, snapping at anything and nothing, until finally paralysis occurs, and he lies on the side of the road, to die in a few hours, if not sooner killed by his pursuers. Sometimes, finding no rest or escape, he goes back to his master's house. But he has undergone an extraordinary change in appearance. He is draggled, and dusty, and exhausted. Ignorant of his true condition, some of the household pity him, and no sooner touch him than they receive the fatal bite.

